# Traumatic Birth Injury in a Term Neonate

**DOI:** 10.7759/cureus.32737

**Published:** 2022-12-20

**Authors:** Andrew Meng, Adarsh Purohith, Austin Huang, Tetiana Litvinchuk

**Affiliations:** 1 Obstetrics and Gynecology, Alabama College of Osteopathic Medicine, Dothan, USA; 2 Pediatrics, Alabama College of Osteopathic Medicine, Dothan, USA; 3 Internal Medicine, Alabama College of Osteopathic Medicine, Dothan, USA; 4 Pediatrics, Helen Keller Hospital, Florence, USA

**Keywords:** arrest of labor, term neonate, extracranial trauma, caput succedaneum, birth injury, cephalohematoma

## Abstract

Cephalohematoma is among the most common forms of birth trauma. It is described as a benign collection of blood above the skull and is associated with prolonged or difficult deliveries. We present the case of a term male born at 39 weeks of gestation with a large cephalohematoma and additional features of caput succedaneum. The patient’s condition was successfully treated with minimal intervention and close observation. This case report is aimed at illustrating an atypical presentation of cephalohematoma and discussing the potential sequelae of extracranial birth trauma.

## Introduction

Extracranial birth injuries of the head are relatively commonplace in labor and delivery. Although frequently benign, severe cases can be potentially detrimental to the health of the neonate [1-5]. Two variations of extracranial birth injury relevant to the present case are cephalohematoma and caput succedaneum.

Cephalohematomas are typically a benign complication of hard labor that is also referred to as subperiosteal hematoma that does not extend beyond suture lines [1]. Although cephalohematomas are frequently unilateral, in some cases, cephalohematomas appear bilaterally. However, in such cases, the edges of both cephalohematomas may be palpated along the sutures [2]. Cephalohematomas develop due to the rupture of emissary or diploic veins and typically emerge due to stresses of labor and delivery, increased cranial pressure in prolonged labor, instrument-assisted delivery, multiple gestation pregnancy, or macrosomia [1,2]. This bleeding is gradual and, thus, develops during the hours or days following birth [1]. In severe cases, bleeding from a damaged emissary vein can pool in the space between the galea aponeurotica and the periosteum; this condition is termed a subgaleal hemorrhage [2]. Patients with cephalohematoma, as well as those with subgaleal hemorrhage, are at an increased risk for hyperbilirubinemia, apnea, and infection [3]. Most cases of cephalohematoma spontaneously and completely resolve within one month, but some are complicated by ossification, calcification, or the formation of a central depression [4,5]. Benign cases of cephalohematoma are often simply observed, while severe cases are managed symptomatically or with surgical excision [4,6].

Caput succedaneum, also typically benign, is described as an edema of the fetal scalp overlying the periosteum that can cross suture lines. Caput succedaneum, similar to cephalohematomas, typically emerges due to the stress of labor and delivery. However, caput succedaneum differs from cephalohematoma in one aspect: it tends to be the largest in size at birth and rapidly resolves within hours to days after birth [2]. Although complications from caput succedaneum are rare, the literature does discuss the potential to elevate bilirubin. Therefore, the management of an infant with caput succedaneum involves observation and routine monitoring for jaundice [7].

We present the case of a term infant male delivered via caesarean section after an arrest of labor. Examination of the infant after birth was notable for a large cephalohematoma and additional features of caput succedaneum. This report examines the unique elements of this case, the importance of rapid neuroimaging, and the risks and benefits of delaying caesarean section in prolonged or arrested labor.

## Case presentation

A male neonate was born at 39 weeks of gestation to a previously G2 T0 P0 A1 L0 diagnosed mother. The mother had presented for prenatal visits and had received typical prenatal care during the pregnancy. Prior to the onset of gestation, she had no underlying medical conditions. During the pregnancy, the mother developed gestational hypertension and third-trimester oligohydramnios. The prenatal ultrasound did not reveal any abnormalities in the fetus. The mother tested positive for group B strep (GBS) at 35 weeks but was determined RPR negative, Rubella immune, and Rh positive. There was no history of maternal or fetal trauma during the pregnancy.

**Figure 1:**
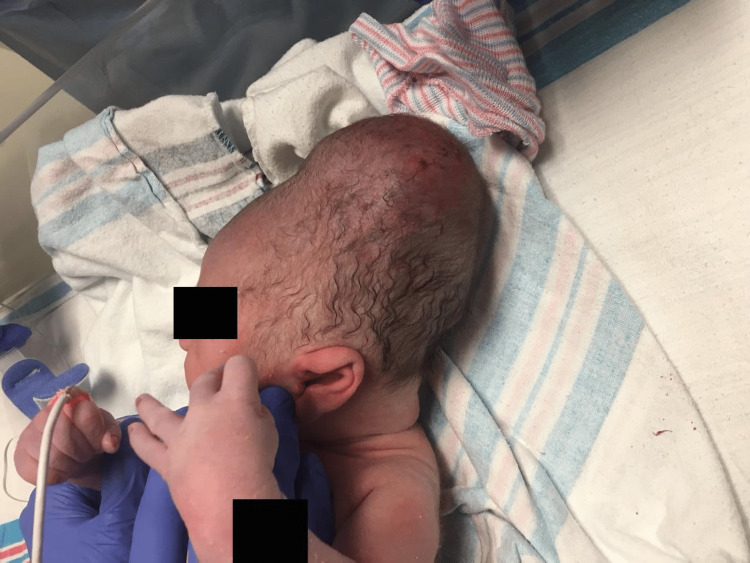
Image of the neonate post-delivery.

At the time of admission for labor, the fetus was in a cephalad position and did not present any condition to contradict vaginal delivery. Vaginal delivery induction was attempted for approximately 24 hours before it was determined that there had been an arrest of labor due to head entrapment of the fetus. An emergent caesarian section was recommended and performed. Upon delivery, the infant was noted to have significant swelling overlying the posterior cranium (Figure 1). The infant weighed 2.965 kg with a head circumference of 32.4 cm. Apgar scores were 2 at one minute, 6 at five minutes, and 8 at 10 minutes. Supportive care was provided, and the neonate was stabilized. Neonate was afebrile, and his heart rate and respiratory rate were within normal ranges. Oxygen saturation was initially low, but it rose to appropriate levels after blow-by oxygen was administered via the nasal cannula. Apart from the aforementioned head injury and a hydrocele, the physical examination did not reveal any abnormality, and the neonate was considered to have an appropriate size for gestational age.

Blood cultures were obtained due to the mother’s GBS-positive status and prolonged labor. The complete blood count was checked and reported to be within normal ranges. A transfontanelle ultrasound of the infant’s brain was performed. Brain parenchyma demonstrated normal echogenicity and the absence of structural abnormality. There was no evidence of intracranial bleeding, and the ventricles appeared to be of normal size. Soft tissue swelling was noted along the posterior scalp, indicating cephalohematoma.

**Figure 2:**
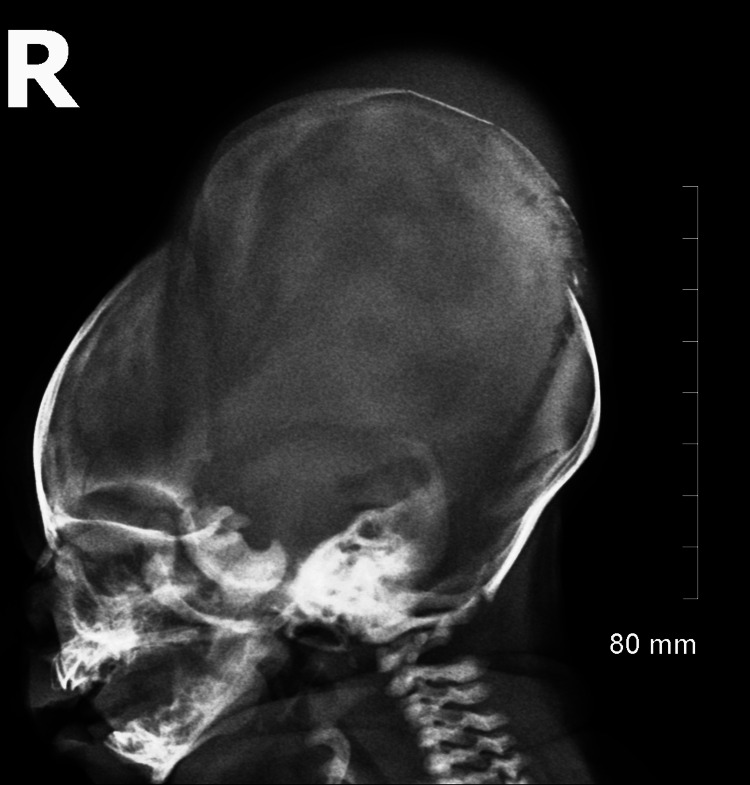
Lateral view x-ray image of the neonate within hours of delivery.

A three-view x-ray of the skull was performed (Figure 2). The imaging revealed an irregular appearance on the parietal calvarium with segmental areas that demonstrated angulation that arose concerns for calvarial fractures. There was also diastases of the sagittal, coronal, and lambdoid sutures. Evidence of cephalohematoma was further extracted through imaging. Although no evidence of intracranial hemorrhage was found, the radiology report recommended further characterization with CT or MR to rule it out.

Due to the need for NICU admission and potential neurosurgical intervention, the infant patient was transferred to a higher-level facility where these resources were available. Upon transfer to the facility, the neonate underwent a head CT without IV contrast. Neuroimaging revealed an area of extra-axial hemorrhage overlaying the left temporal lobe and a small intraventricular hemorrhage. No evidence of fracture was found. Neurosurgical intervention was not recommended.

The neonate was weaned off the nasal cannula to room air and was able to maintain appropriate oxygen saturation. Due to the increased risk of GBS infection, the neonate was started on IV ampicillin 145mg q8H. Additional laboratory tests were ordered, and the lab results are displayed in Table 1.

**Table 1:** Routine lab results obtained upon admission. TSH: thyroid stimulating hormone; Free T4: thyroxine; BUN: blood urea nitrogen; CO2: bicarbonate The Chem10 panel comprises sodium, chloride, BUN, potassium, CO2, creatinine, glucose, calcium, phosphate, and albumin.

Lab finding	Day one value	Day one interpretation	Day two value	Day 2 Interpretation
Total bilirubin	5.20 mg/dL	Normal	9.81 mg/dL	Elevated
Direct bilirubin	0.30 mg/dL	Normal	0.42 mg/dL	Normal
Indirect bilirubin	4.90 mg/dL	Normal	9.39 mg/dL	Elevated
TSH	21.35 mIU/L	Elevated	9.35 mIU/L	Normal
Free T4	1.53 ng/dL	Normal	2.90 ng/dL	Normal
Sodium	132 mmol/L	Normal	134 mmol/L	Normal
Chloride	100 mmol/L	Normal	104 mmol/L	Normal
BUN	4 mg/dL	Normal	6 mg/dL	Normal
Potassium	4.1 mmol/L	Normal	3.6 mmol/L	Normal
CO2	18 mmol/L	Normal	17 mmol/L	Normal
Creatinine	0.66 mg/dL	Normal	0.84 mg/dL	Normal
Glucose	79 mg/dL	Normal	67 mg/dL	Normal
Calcium	9.3 mg/dL	Normal	8.9 mg/dL	Normal
Phosphate	5.4 mg/dL	Normal	4.7 mg/dL	Normal
Albumin	3.7 g/dl	Normal	3.6 g/dl	Normal
Hematocrit	42.7%	Normal	41.9%	Normal

Day one lab values demonstrated an elevated thyroid stimulating hormone (TSH) level with normal thyroxine (Free T4), bilirubin, Chem10 panel, and hematocrit. Day four values demonstrated consistently normal results for the Chem10 panel and hematocrit and an improved TSH. However, free T4 had significantly increased, and unconjugated bilirubin had increased above the normal range. Endocrinology was consulted on the neonate’s thyroid function. The impression was that the TSH was initially elevated due to fetal birth trauma and that the free T4 was appropriately elevated due to normal physiological pathways. Previously drawn blood cultures revealed no bacterial growth, and the administration of antibiotics was discontinued after 36 hours.

The neonate was determined to be in stable condition and discharged on post-natal day two. Weight at discharge was 2.971 kg and head circumference measured at 33 cm. A follow-up appointment with the primary care provider was scheduled to check on the thyroid and bilirubin levels and to administer routine newborn care.

**Figure 3:**
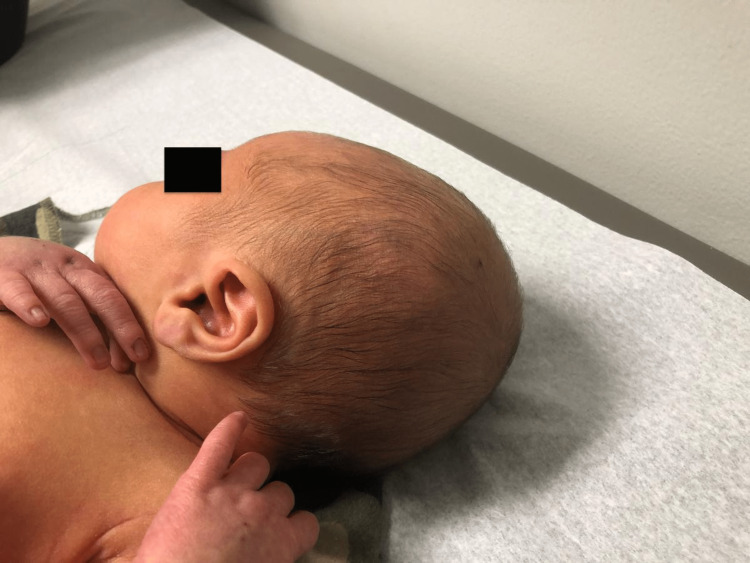
Image of the neonate on post-natal day four.

On post-natal day four, the neonate presented to the pediatrician’s office for an initial wellness visit. The parents noted that the infant was active and had no difficulty with receiving feedings. An examination showed significantly reduced soft tissue swelling at the vertex of the head and no erythema (Figure 3). On further examination, mild jaundice extending from the head to the chest was revealed. A subsequent bilirubin measurement was ordered, which revealed an indirect bilirubin level of 13 mg/dL. All other physical exam findings were within normal limits. TSH measured 3.5 mIU/L and free T4 measured 3.26 ng/dL, indicating a continued improvement of thyroid function. On post-natal day six, the additional indirect bilirubin level was 10.2 mg/dl, demonstrating an improvement in bilirubin metabolism.

The patient appears to have fully recovered from his birth injury without any long-term complications. Currently, there is no evidence that his initial condition will negatively affect his health or development.

## Discussion

The patient’s primary diagnosis was cephalohematoma. However, significant aspects of his presentation were atypical of cephalohematomas. First, while a typical cephalohematoma develops over hours or days after delivery, this patient’s cephalohematoma appeared to have developed at the time of delivery [2]. Second, the literature describes cephalohematomas as benign and non-emergent; however, the neonate was in distress immediately following delivery [1]. Third, cephalohematomas are often described as unilateral masses, but the patient’s lesion was apparent bilaterally [1]. Fourth, the literature describes cephalohematomas as persisting for weeks or months, which is inconsistent with our case where marked resolution of the cephalohematoma occurred within four days of delivery [2].

Interestingly, three of the atypical elements observed in this case are typical of caput succedaneum: onset at birth, a bilateral lesion, and rapid resolution [7]. While our patient may have solely had an atypical presentation of cephalohematoma, another possibility is concomitant caput succedaneum. The literature has not specifically described both conditions occurring together; however, with similar risk factors and different mechanisms of pathogenesis, it is possible for them to present simultaneously.

Although it frequently occurs with cephalohematomas, the presentation of jaundice at the newborn visit was another notable aspect of this case. Physiologic jaundice is the dermatological manifestation of neonatal hyperbilirubinemia. Newborn infants are known to have increased total serum bilirubin compared to adults due to shorter red blood cell lifespan and immature metabolism [8]. The extravascular pooling of blood in a cephalohematoma may explain the increased bilirubin load and resultant jaundice in this patient. When neonatal jaundice is observed in combination with a cephalohematoma, close observation of bilirubin levels should be implemented to monitor the development of kernicterus and associated neurological sequelae [1]. While cephalohematoma can be the cause of hyperbilirubinemia and jaundice in a neonate, other causes such as feeding difficulties, genetic enzyme deficiencies, or hemoglobinopathies should also be considered.

While the cause of cephalohematomas is multifactorial, early recognition and management of risk factors could minimize its occurrence. As previously mentioned, cephalohematomas frequently occur due to stresses of labor and delivery, increased cranial pressure in prolonged labor, instrument-assisted delivery, multiple gestation pregnancy, or macrosomia [1,2]. Careful prenatal and peripartum assessment can identify these threats before birth trauma occurs or worsens in severity [2]. Evaluation tools such as ultrasound, pelvic exam, and electronic fetal monitoring are often sufficient to alert the physician to circumstances of elevated risks and facilitate early intervention [2].

When cephalohematoma is noted, proper evaluation is essential to monitor its progression and any complications. As the presentation and complications of cephalohematoma are varied, the literature recommends symptom-based management [1,2]. A typical, benign cephalohematoma is often clinically diagnosed and managed primarily with observation; however, radiological exams may be used to rule out further injury to the region [1]. In our case, the neonate was appropriately assessed and monitored after the characteristic bulge was noted. The combination of a low APGAR score and extensive swelling of the cranium provoked suspicion of a subgaleal hemorrhage, which, if unrecognized, could lead to severe hypotension and a significant risk of morbidity [2]. Consequently, rapid neuroimaging was of immense importance in ensuring that the care provided was sufficient and timely. Incidentally, an intraventricular hemorrhage was observed with CT imaging.

Intraventricular hemorrhage could potentially lead to death or permanent neurological damage; therefore, this finding highlights the need to monitor for worsening lethargy or irritability [2]. While this patient’s presentation indicated a need for imaging, similar radiological interventions may not be appropriate in other cases. Radiation exposure is always a risk factor, which must be considered, especially in the younger population. In early life, a high proportion of cells undergo rapid division, and radiation exposure significantly increases the risk of significant DNA damage and eventual cancer development [9]. Most imaging techniques utilized in standard NICUs follow low radiation dose recommendations; however, the intrinsic risk must be properly weighed against the need for imaging [10].

## Conclusions

Considering the commonality of cephalohematomas and the possibilities of sequelae, prompt and appropriate evaluation and management of the condition are important throughout the pregnancy, labor, delivery, and postnatal periods. Various complications have been reported in the literature; however, most cases follow a benign course and spontaneously reabsorb. We presented the case of an infant presenting with a large cephalohematoma after delivery; the cephalohematoma spontaneously resolved within four days without any major complications or specific interventions aside from monitoring. We hope that this case will aid in the evaluation and management of similar cases in the future.
